# The transplantation of the gut microbiome of fat-1 mice protects against colonic mucus layer disruption and endoplasmic reticulum stress induced by high fat diet

**DOI:** 10.1080/19490976.2024.2356270

**Published:** 2024-05-26

**Authors:** Amina Bourragat, Quentin Escoula, Sandrine Bellenger, Olivier Zemb, Martin Beaumont, Killian Chaumonnot, Jean-Pierre Farine, Emmanuel Jacotot, Aline Bonnotte, Laure Avoscan, Jeanine Lherminier, Kangjia Luo, Michel Narce, Jérôme Bellenger

**Affiliations:** a CTM UMR1231, Université de Bourgogne, Dijon, France; b CTM UMR1231, INSERM, Dijon, France; c LipSTIC LabEx, FCS Bourgogne-Franche Comté, Dijon, France; d Valorex, La Messayais, Combourtillé, France; e GenPhySE, Université de Toulouse, INRAE, ENVT, Castanet-Tolosan, France; f Centre des Sciences du Goût et de l’Alimentation, UMR6265 CNRS, UMR1324 INRA, Université de Bourgogne, Dijon, France; g L’Institut Agro Dijon, PAM UMR A 02.102, Université de Bourgogne, Dijon, France; h Agroécologie, L’Institut Agro Dijon, CNRS, INRAE, Plateforme DimaCell, Dijon, France

**Keywords:** Obesity, mucus layer- ER stress, gut permeability, omega-3 polyunsaturated fatty acids, microbiome transplantation

## Abstract

High-fat diets alter gut barrier integrity, leading to endotoxemia by impacting epithelial functions and inducing endoplasmic reticulum (ER) stress in intestinal secretory goblet cells. Indeed, ER stress, which is an important contributor to many chronic diseases such as obesity and obesity-related disorders, leads to altered synthesis and secretion of mucins that form the protective mucus barrier. In the present study, we investigated the relative contribution of omega-3 polyunsaturated fatty acid (PUFAs)-modified microbiota to alleviating alterations in intestinal mucus layer thickness and preserving gut barrier integrity. Male fat-1 transgenic mice (exhibiting endogenous omega-3 PUFAs tissue enrichment) and wild-type (WT) littermates were fed either an obesogenic high-fat diet (HFD) or a control diet. Unlike WT mice, HFD-fed fat-1 mice were protected against mucus layer alterations as well as an ER stress-mediated decrease in mucin expression. Moreover, cecal microbiota transferred from fat-1 to WT mice prevented changes in the colonic mucus layer mainly through colonic ER stress downregulation. These findings highlight a novel feature of the preventive effects of omega-3 fatty acids against intestinal permeability in obesity-related conditions.

## Introduction

Obesity, now considered a real worldwide epidemic affecting more than 650 million people, is complex and mainly associated with excessive energy intake and changes in dietary habits favoring the consumption of diets high in saturated fat and sugar.^1^ This multifactorial pathology is linked to chronic low-grade systemic inflammation, contributing to the development of chronic disorders, such as diabetes, nonalcoholic fatty liver, and cardiovascular diseases.^2^

A high-fat diet (HFD) has been shown to increase intestinal permeability, which is partly attributed to the downregulation of genes encoding tight junctions such as zonula-occludens 1 and occludin,^3^ which modestly increase circulating levels of bacterial lipopolysaccharides (LPS), called metabolic endotoxemia, in response to noninfectious stimuli.^4^ LPS then binds to Toll-like receptor 4, which initiates an intracellular signaling cascade, resulting in the downstream activation of inflammatory signaling pathways.^5^ Metabolic endotoxemia has been shown to be closely related to intestinal microbiota dysbiosis during HF feeding both in mice^6^ and humans.^7^ Thus, the gut microbiome has emerged as a culprit in the development of chronic diseases such as obesity^8,9^ and diabetes.^10,11^

A dense physical mucus barrier covering the gut epithelial cell surface has long been considered a simple lubricant for facilitating the progression of stools. The regulation of this neglected component of gut barrier function is now gaining increasing attention^12^ as it prevents the translocation of commensal and pathogenic microorganisms across the intestinal epithelium and then protects the host against injuries.^13^ The colonic mucus barrier is organized in two layers: an inner, stratified, firmly adherent to the epithelial cells that does not allow bacteria to penetrate, and an outer, loose, and thicker layer, where commensal bacteria live and thrive.^14^ The glycoprotein mucin 2 (MUC2), secreted by intestinal goblet cells, is the predominant component of the colonic mucus layers.^15^ MUC2 contains cysteine-rich and highly O-glycosylated domains that require post-translational modifications in endoplasmic reticulum (ER) and Golgi.^14^ In the context of obesity, HFD leads to colonic mucus layer disruptions,^16^ which consequently increase intestinal permeability and LPS leakage.

The glycoprotein MUC2 is susceptible to unfolding/misfolding, which can initiate ER stress and inflammation under stress conditions.^17^ Moreover, altered MUC2 protein expression in the colon of HFD-fed mice has been associated with misfolding of the protein in the ER and increased intestinal expression of ER stress markers, detrimentally affecting production of the secreted mucosal barrier and increasing intestinal epithelial paracellular permeability.^18^ ER stress is an important contributor to many chronic diseases such as obesity and obesity-related disorders.^19–22^ In addition, saturated fatty acids (*e.g*., palmitic acid, PAL, high in HFD) have been shown to be powerful activators of ER stress in many cells, including pancreatic β-cells, muscle cells, hepatocytes and goblet cells.^23–26^

Omega-3 polyunsaturated fatty acids (PUFAs) are known for their powerful anti-inflammatory, anti-diabetic^27^ and anti-obesity properties, as we recently showed in HFD-fed fat-1 mice, which are linked to microbiota modulation.^3^ Indeed, omega-3-modified microbiomes transplanted into wild-type mice were able to prevent weight gain and normalize intestinal permeability.^3^ Gut microbiota transplantation has emerged as a promising and successful intervention for managing obesity and intestinal inflammatory disorders in both animals and humans.^28^ Moreover, omega-3-PUFAs also prevent PAL-induced ER stress in numerous cellular models.^29–31^ We recently showed that PAL-altered MUC2 production in LS174T cells was alleviated by omega-3 PUFAs and that such prevention was partly mediated through ER stress downregulation.^26^ Nevertheless, the relevance of such *in vitro* data remains to be explored *in vivo* in the context of dietary obesity, as well as the efficiency of omega-3 PUFAs in alleviating colonic mucus layer alterations and consequently preserving the gut barrier integrity. Then, after studying the impact of omega-3 PUFAs tissue enrichment along the mice gastrointestinal tract on obesity-related metabolic disorders and on intestinal epithelium integrity in HFD conditions,^3^ we now propose to evaluate its impact on the mucus layer, another key component of gut barrier function. For that, as previously,^3,32^ we took advantage of fat-1 mice to study the effect of gut microbiome alteration *versus* host tissue fatty acid composition on the metabolic phenotype in mice fed an HF diet. The fat-1 transgenic mice encoding an omega-3 fatty acid desaturase (*fat-1* gene from the roundworm *Caenorhabditis elegans*) are able to catalyze the conversion of omega-6-to-omega-3 PUFAs within all their tissues,^33^ overcoming the use of dietary manipulation. Compared with conventional dietary intervention (introducing potential confounding factors), this approach is more effective in balancing the omega-6-to-omega-3 ratio because it not only elevates tissue concentrations of omega-3 PUFAs but also decreases the levels of excessive endogenous omega-6 PUFAs,^33^ which is ideal for identifying the specific roles of omega-3 PUFAs and addressing nutrient-gene interactions. Then, this mouse model represents a useful *in vivo* system for giving new insights of the role of the omega-6-to-omega-3 fatty acid ratio in the context of obesity and associated metabolic disorders.

In the present study, we show for the first time that omega-3 PUFAs tissue enrichment preserves the thickness and structure of the colonic mucus layer through ER stress downregulation under HFD conditions. Similar results were obtained after omega-3-modified microbiota transplantation into HFD-fed wild-type mice, providing evidence that this microbiota accounts for the protective effects observed in diet-induced obesity and associated metabolic disorders. These findings highlight a novel aspect of the preventive effects of omega-3 fatty acids against intestinal disorders that occur under obesity-related conditions.

## Results

### HFD-fed fat-1 mice are protected against colonic mucus layer alteration (Figure 1)

**Figure 1.**
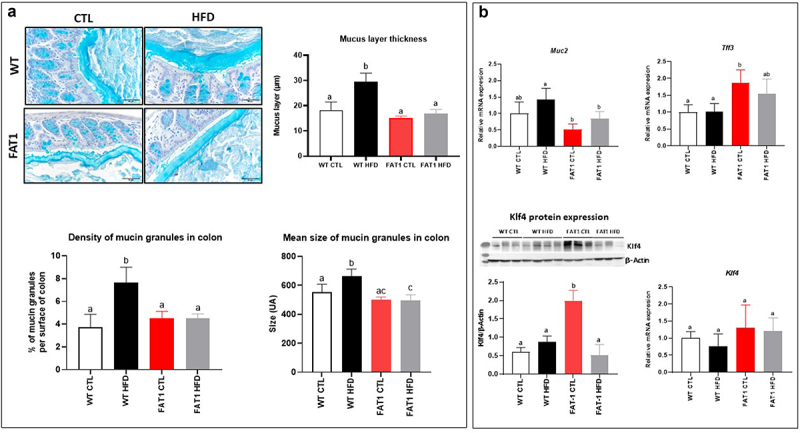
HFD-fed fat-1 mice are protected against colonic mucus layer alteration.

Previous studies have reported changes in the thickness of the colonic mucus layer in mice fed a HFD.^18,34,35^ As shown in Figure 1(a), we observed in our experimental conditions that the mucus layer of wild-type (WT) mice fed a HFD was nearly twice as thick as that of control (CTL) diet-fed mice. In contrast, we demonstrated for the first time that omega-3 PUFAs tissue enrichment completely prevented the alteration of the mucus layer in fat-1 mice fed the HFD, as the thickness of the mucus layer remained comparable to values measured in WT and fat-1 animals fed the CTL diet. Density and mean size of the mucin granules in the colon of the mice (Figure 1(a)) have been performed and we evidenced these parameters to be higher in HFD-fed wild-type mice compared with CTL-fed wild-type mice. In fat-1 mice (whatever the diet), the density and mean size of mucin granules remained comparable to those of CTL-fed wild-type animals. In addition, Muc2 gene expression was reduced in fat-1 (FAT1) HFD when compared to that in WT HFD mice. Moreover, whereas no change in *Klf4* (Kruppel-like factor 4, a goblet cell differentiation factor) gene expression was observed, its protein expression was significantly increased in FAT1 CTL group compared with WT groups and HFD-fed fat-1 mice. In addition, *Tff3* (Trefoil factor 3, important for gut mucosal protection and restitution) was increased in FAT1 CTL mice compared with WT animals (Figure 1(b)).

Mice (fat-1 transgenic and WT littermates) were fed CTL or HF diet for 11 weeks. a: Representative photographs of Carnoy-fixed colonic tissue sections stained with Alcian blue/periodic acid-Schiff from WT (*top*) and fat-1 (*bottom*) mice fed a CTL or HF diet (*n*=8 per group) that were used for mucus layer thickness measurements (scale bars, 100 µm). b: Colonic mRNA expression of *Muc2*, goblet cell differentiation factor *Klf4* and *Tff3* and KLF4 protein expression, involved in mucosal protection and thickening of the mucus (*n*=8 per group). Data are shown as mean ± SEM, and differences were analyzed by Tukey’s multiple comparison test. Bars assigned different superscript letters (a, b, c) are statistically different at *p*<0.05. MUC-2: mucin 2. KLF4: Krüppel-like factor 4. TFF3: Trefoil factor of 3.

### Omega-3 tissue enrichment protects fat-1 mice against HFD-induced structure changes of the colonic mucus layer (Figure 2)

Beyond the study of the thickness of the mucus layer, we considered that a better understanding of its architecture would help evaluate the impact of omega-3 enrichment in such altered thickness prevention. We then performed electron microscopy on the colon sections to study the complex and highly structured organization of the colon mucus gel.

**Figure 2.**
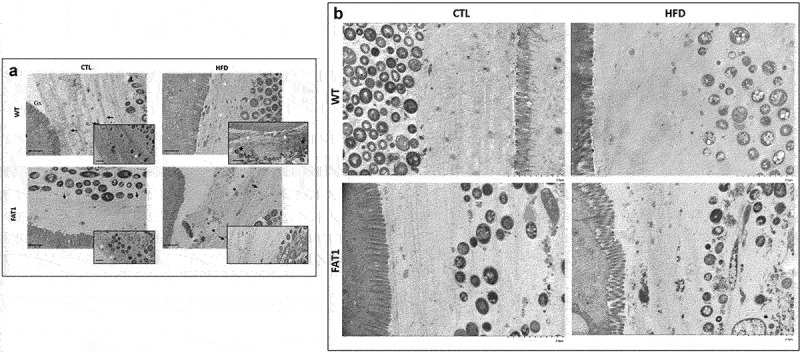
Omega-3 tissue enrichment protects fat-1 mice against HFD-induced structure changes of the colonic mucus layer.

The colons of CTL diet-fed WT mice displayed a highly stratified inner mucus layer, compact and multi-layers were visible (arrow inset images), while the mucus layer at the level of the microbiota was looser (Figure 2(a)). Some isolated bacteria were present with relatively intact cells toward the outer layers (white asterisk inset image) and more degraded cellular material toward the inner layers (black asterisk inset image). The glycocalyx appeared electron-dense and was mainly distributed on the tip of the microvilli.

Mice (fat-1 transgenic and WT littermates) were fed CTL or HF diet for 11 weeks. a: Representative electron microscopy images of colons from WT and fat-1 mice (three mice per group) fed a control (CTL) or a high-fat diet (HFD). The enlarged images of the second row are from a part of the first row (scale bar, 1 mm). b: Electron microscopy images (Magnification =X50.0k) of WT and FAT1 mice (three mice per group) fed a control (CTL) or a high-fat diet (HFD).

In contrast to CTL diet-fed WT mice, HFD-fed WT animals exhibited a more heterogeneous and looser mucus structure, favoring a probable escape of bacteria toward the inner layers (black stars inset image). These bacteria are bordered by a smaller stratified zone (white arrows).

A clearly distinct and stratified bacteria-limiting zone (black arrows) was observed, and the mucus mesh appeared looser outside this area in the colon of CTL diet-fed transgenic mice (Figure 2(a)). Some bacteria were isolated within stratified mucus (white asterisks, inset image).

Unlike what has been observed in HFD-fed WT mice, the colon of fat-1 mice fed with HFD showed a highly stratified outer mucus layer, keeping bacteria away from epithelial cells (black arrows), and punctuated by uncharacterized pockets (black asterisks). In order to provide stronger evidence of the protection of the HFD-fed fat-1 mice against altered colonic mucus layer structure, electron microscopy images (Magnification =×50.0k) were performed (Figure 2(b)). In the CTL-fed wild-type, CTL-fed fat-1, and HF-fed fat-1 mice the MUC2 mucin-dependent mucus layer in colon is characterized by a dense well organized stratified lamellar appearance. In contrast, HF-fed wild-type animals exhibit a less dense (loose) mucin net-like structure (Figure 2(b)).

### Markers of the colonic endoplasmic reticulum stress are alleviated in HFD-fed fat-1 mice (Figure 3)

**Figure 3.**
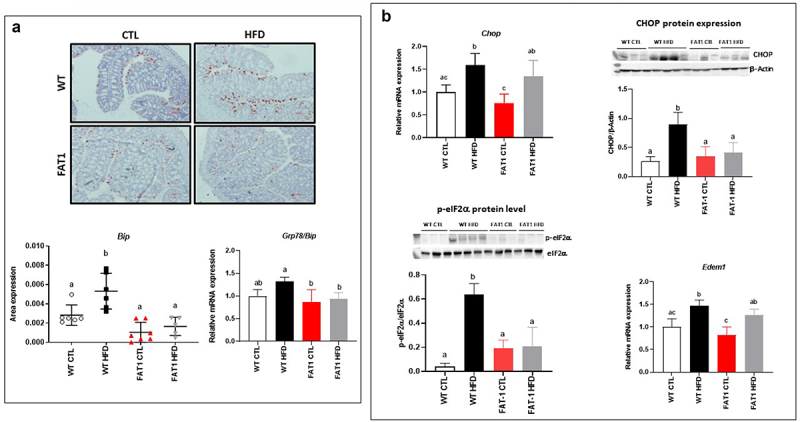
Markers of the colonic endoplasmic reticulum stress are alleviated in HFD-fed fat-1 mice.

Mucins, large glycoproteins that require many post-translational modifications, are susceptible to unfolding/misfolding, which can initiate ER stress,^17^ an important contributor to many chronic diseases such as obesity and obesity-related disorders.^19,20^ In accordance with these observations, we reported here that HFD increased colon immunolabelling of the central regulator of ER stress because of its role as a major ER chaperone, BiP (immunoglobulin heavy chain binding protein), in WT mice. However, HFD-fed fat-1 mice exhibited immunostaining consistent with that observed in CTL diet-fed animals (Figure 3(a)). In line with BiP levels, colonic mRNA expression of several genes involved in ER stress, such as *Chop* (C/EBP homologous protein) and *Edem 1* (ER-degradation-enhancing alpha-mannosidase-like protein-1), was increased in WT mice fed with the obesogenic diet, while it remained similar to CTL-fed animals in HFD-fed fat-1 mice for *Grp* (glucose-regulated protein) *78*. In addition, the protein expressions of CHOP and phosphorylated eIF2α (Eukaryotic initiation factor 2α) were significantly increased in HFD-fed WT mice when they remained comparable to CTL-fed WT mice in fat-1 animals whatever the diet (Figure 3(b)).

Mice (fat-1 transgenic and WT littermates) were fed CTL or HF diet for 11 weeks. a: Representative photographs (20X objective) of immunohistochemistry for BiP in the distal colon of WT (*top*) and fat-1 mice (*bottom*) fed CTL or HF diet. Colonic mRNA expression of *GRP78/Bip*. b: Colonic mRNA expression of *Chop*, *Edem1* and colonic protein expressions of CHOP, eIF2α and Phospho-eIF2α (Ser51) (*n* = 8 per group). Data are shown as mean ± SEM, and differences were analyzed by Tukey’s multiple comparison test. Bars assigned different superscript letters (a, b, c) are statistically different at *p* < 0.05. BiP: Binding immunoglobulin protein. GRP78: glucose-regulated protein 78. CHOP: C/EBP Homologous Protein. Edem1: ER degradation enhancer, mannosidase alpha-like 1. eIF2α: eukaryotic initiation factor 2.

### Impact of high-fat feeding and omega-3 fatty acid tissue enrichment on colonic autophagy (Figure 4)

**Figure 4.**
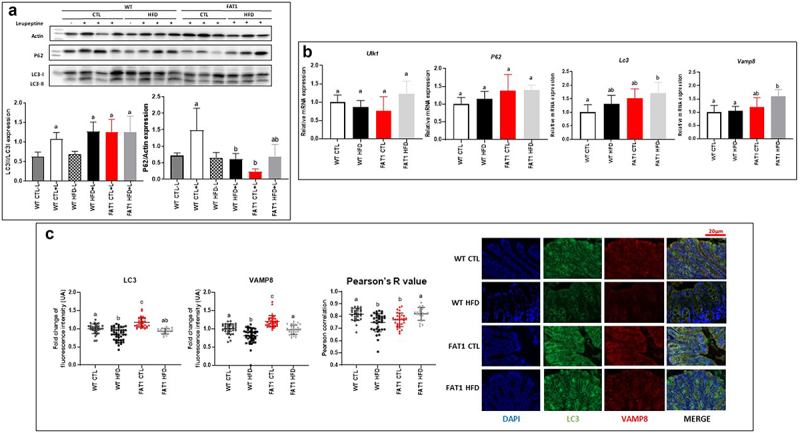
Impact of high fat feeding and omega-3 fatty acid tissue enrichment on colonic autophagy.

In addition to synthesizing MUC2, goblet cells release stored MUC2 granules in response to various stimuli. Secretory autophagy has emerged as an alternative non-degradative mechanism for cellular trafficking and unconventional protein secretion.^36,37^ Indeed, work by Patel *et al*. has shown that autophagy is required for mucin granule secretion, as mice lacking *Atg* (Autophagy related) *5* are impaired in mucin secretion, which leads to larger goblet cells than those seen in their wild-type counterparts when goblet cell numbers remain unaffected.^37^ We then examined LC3 (Microtubule-associated protein 1A/1B-light chain 3) and P62 (Sequestosome-1) protein expression (key proteins of autophagic flux) and a number of known autophagy markers using qPCR. P62 protein expression was decreased by HFD and omega-3 tissue enrichment, whereas neither the diet nor the genotype modified the expression of LC3 (Figure 4(a)). Gene expression of autophagy (*Ulk1*(unc51-like kinase-1), *P62 and* LC3) and exocytosis (*Vamp8*, Vesicle-associated membrane protein 8) markers was not modified by HFD in WT mice. Only LC3 and Vamp8 expression increased in FAT1 HFD *versus* WT CTL. Nevertheless, gene expression of autophagy and exocytosis markers were not modified by HFD in fat-1 mice (Figure 4(b)). As coordinated mucus exocytosis maintains homeostasis in the intestinal epithelium, we evaluated the impact of the HFD on the expression of the SNARE (Soluble N-ethylmaleimide-sensitive-factor Attachment protein Receptor) protein VAMP8, which coordinates mucin exocytosis from goblet cells.^38^ LC3 and Vamp8 protein colocalization showed that mucin exocytosis seemed to be altered by HFD in WT mice only (Figure 4(c)).

Mice (fat-1 transgenic and WT littermates) were fed CTL or HF diet for 11 weeks. a: Expression of LC3 in the colon tissue from WT and fat-1 mice fed CTL or HF diet by western blot analysis. b: Relative mRNA expression of *Ulk1*, *P62*, LC3 and *Vamp8* in the colon tissue of mice as detected by qPCR. c: Representative images of LC3 and VAMP8 immunofluorescence in colon sections of WT and fat-1 mice fed the CTL or HF diet (Scale bar 20 µm). Colocalization of Vamp8 and LC3 within tissue sections was assessed from ROIs within the epithelium and calculated for the Pearson correlation coefficient with a value of 1 indicating perfect colocalization and 0 indicating no colocalization (six mice per group, >10 ROIs per mouse). Data are shown as mean ± SEM, and differences were analyzed by Tukey’s multiple comparison test. Bars assigned different superscript letters (a, b, c) are statistically different at *p* < 0.05. ULK1: unc51-like kinase-1. P62: Sequestosome-1. LC3: Microtubule-associated protein 1A/1B-light chain 3. VAMP8: Vesicle-associated membrane protein 8.

### Transplantation of fat-1 microbiome decreases weight gain, enhances metabolic parameters and alleviates intestinal alteration in mice fed a HFD (Figure 5)

**Figure 5.**
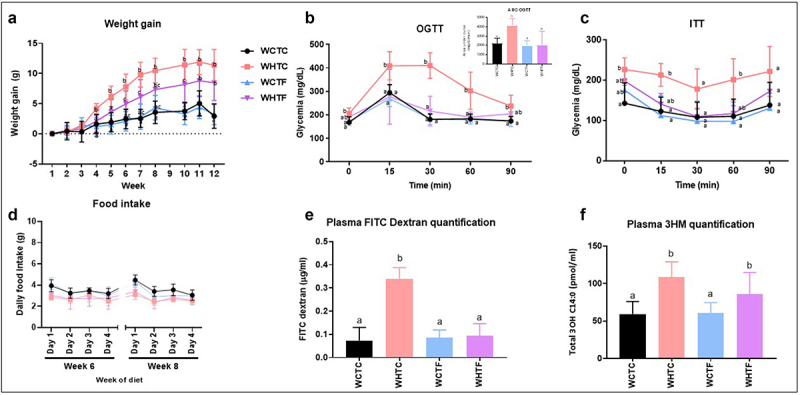
Transplantation of fat-1 microbiome decreases weight gain, enhances metabolic parameters and alleviates intestinal alteration in mice fed a HFD.

We and others have recently shown that the ability of the gut microbiota to modulate obesity can be transferred to other animals.^32,39^ To determine whether the gut microbiome of omega-3 PUFAs-enriched mice may improve obesity-related traits in HFD-fed mice, the cecal microbiome of fat-1 mice was transferred to WT mice fed a CTL or HFD without any prior antibiotic treatment. Before that, we previously analyzed the gut microbiota profile in wild-type and fat-1 mice (Supplemental Figures S4 and S5) and showed the relatively consistent composition of the inocula over time. We highlighted variations in *Odoribacter*, *Parabacteroides*, *Rombustia*, *Oscillibacter*, and *Intestimonas*, even though these differences did not meet the false discovery rate (FDR) correction threshold. Moreover, fat-1 microbiome exhibited higher abundance of *Butyricicoccus*, *Prevotellaceae_UCG-001*, *Peptococcus*, and *Bifidobacterium* than wild-type microbiome. While no difference in food intake was observed between WT-to-WT and fat-1-to-WT mice fed the CTL or HFD (Figure 5(d)), HFD-fed WT mice transplanted with the fat-1 microbiome exhibited a lower weight gain than the obese WT counterparts force-fed with the WT microbiome (9.2 ± 1.7 versus 13.0 ± 1.8, respectively) (Figure 5(a)) Nevertheless, on weeks 11 and 12, this difference became non-significant between the two groups. Moreover, a significantly improved glucose homeostasis was observed in HFD-fed WT mice transplanted with fat-1 microbiome (Figures 5(b,c)). We and others have already shown that HFD alters gut permeability, leading to bacterial component flux such as LPS.^3,40^ Twelve weeks of high fat diet increased gut permeability of WT mice transplanted with WT microbiome, whereas DX-FITC (Fluorescein Isothiocyanate-Dextran) gut permeability of HF-fed WT mice transplanted with fat-1 microbiome remained similar to that of control animals (Figure 5(d)). In addition, plasma LPS measurements revealed increased endotoxemia in HFD-fed animals, regardless of the transplanted microbiome (Figure 5(f)).

WT mice were transplanted with the microbiome of WT or fat-1 mice and fed CTL or HF diet for 12 weeks. a: Weight gain curves (*n* = 8 per group). b: OGTT and (inset) mean area under the curve (AUC) measured between 0 and 120 min after glucose loading (*n* = 6 per group). c: ITT measured between 0 and 90 min after glucose loading (*n* = 6 per group). d: Food intake has been carried out at 6 and 8 weeks of the feeding period (*n* = 8 per group). e: Intestinal permeability assay: Plasma FITC-dextran 4 (DX, 4,000 molecular weight) oral challenge was measured in mice (*n* = 8 per group) fed CTL or HF diet for 12 weeks. f: Direct plasma quantitation of 3-β-hydroxymyristic (OH) acid concentration by gas chromatography-mass spectrometry in mice fed CTL or HF diet for 12 weeks (*n* = 8 per group). Data are shown as mean ± SEM, and differences were analyzed by Tukey’s multiple comparison test. Data with different superscript letters (a, b, c) are significantly different at *p* < 0.05. WTW CTL: WT mice transplanted with microbiota of WT mice fed a CTL diet, WTW HFD: WT mice transplanted with microbiota of WT mice fed a HFD, WTF CTL: WT mice transplanted with microbiota of fat-1 mice fed a CTL diet, and WTF HFD: WT mice transplanted with microbiota of fat-1 mice fed a HFD. OGTT: Oral Glucose Tolerance Test. ITT: Insulin Tolerance Test.

### Transplantation of the fat-1 microbiome prevents the increase of the thickness of the colonic mucus layer in mice fed the HF diet (Figure 6)

**Figure 6.**
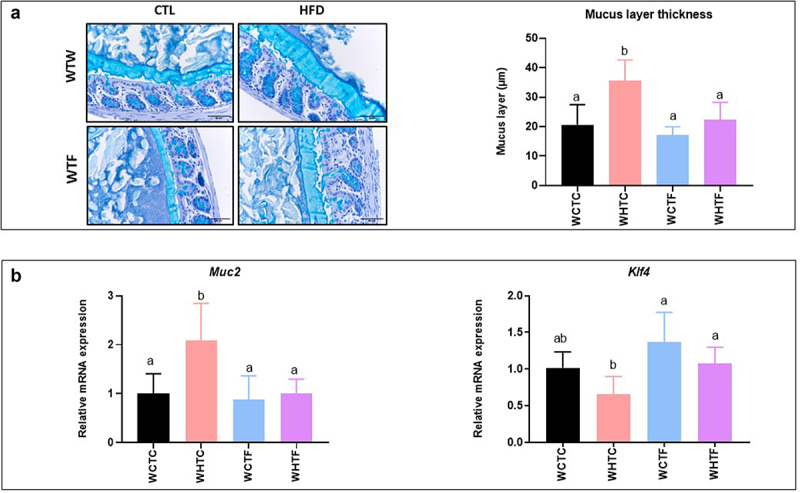
Transplantation of the fat-1 microbiome prevents the increase of the thickness of the colonic mucus layer in mice fed the HF diet.

To study the relative contribution of microbiota, a key element of the gut barrier function, on the colonic mucus layer of HFD-fed mice, we evaluated the impact of multiple transfers of the microbiome on the morphology of the colonic mucus layer. Transplanting the microbiome of WT mice confirmed an increase in mucus layer thickness in HFD-fed WT recipient mice. However, the thickness of the mucus layer of WT recipient mice that had received the fat-1 microbiome and were fed the HF diet remained similar to that measured in control animals (Figure 6(a)). Moreover, colon *Muc2* gene expression (the major protein secreted by goblet cells) significantly increased in HFD-fed mice transplanted with the WT microbiome, but remained unaffected when the fat-1 microbiome was used. Finally, the expression of the zinc-finger transcription factor *Klf4* tended to decrease in HFD-fed mice transplanted with the WT microbiome, whereas its expression remained similar to controls in HFD-fed WT mice transplanted with the fat-1 microbiome (Figure 6(b)).

WT mice were transplanted with the microbiome of WT or fat-1 mice and fed CTL or HF diet for 12 weeks. a: Representative photographs of Carnoy-fixed colonic tissue sections stained with Alcian blue/periodic acid-Schiff of mice transplanted with the microbiome of WT or fat-1 mice and fed a CTL or HF diet (*n* = 8 per group) used for mucus layer thickness measurements (scale bars, 100 µm). b: Colonic mRNA expression of *Muc2* and *Klf4* (*n* = 8 per group). Data are shown as mean ± SEM, and differences were analyzed by Tukey’s multiple comparison test. Bars assigned different superscript letters (a, b, c) are statistically different at *p* < 0.05. WTW CTL: WT mice transplanted with microbiota of WT mice fed a CTL diet, WTW HFD: WT mice transplanted with microbiota of WT mice fed a HFD, WTF CTL: WT mice transplanted with microbiota of fat-1 mice fed a CTL diet, and WTF HFD: WT mice transplanted with microbiota of fat-1 mice fed a HFD. MUC2: mucin 2. KLF4: Krüppel-like factor 4.

### Transplanting fat-1 microbiome protects HFD-fed WT mice against colonic endoplasmic reticulum stress (Figure 7)

**Figure 7.**
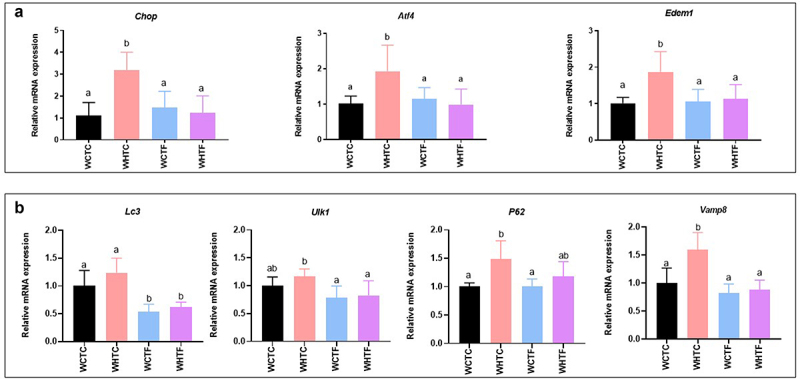
Transplanting fat-1 microbiome protects HFD-fed WT mice against colonic endoplasmic reticulum stress.

Since enrichment in omega-3 PUFAs prevents HFD-fed fat-1 mice against colonic ER stress, we investigated the involvement of the fat-1 microbiome in ER stress prevention when transplanted into HFD-fed WT mice. As expected, after 12 weeks of HFD, WT mice transplanted with the WT microbiome exhibited a two-fold increase in colonic mRNA expression of several ER stress markers such as *Chop*, *Atf* (Activating transcription factor) *4* and *Edem1*. However, the fat-1 microbiome transplanted into HFD-fed WT mice completely prevented this overexpression. Indeed, these expression levels remained similar to those observed in the control group (Figure 7(a)). The study of early markers of autophagy showed, under our experimental conditions, that the transfer of WT microbiome to WT mice fed HFD did not change *Lc3* and *Ulk1* gene expression. Regarding the mucus secretion process, the expression of *Vamp8* and *P62* was significantly higher in WHTC than in WCTC. Regardless of diet, the transfer of the fat-1 microbiome decreased *Lc3*, *Ulk1* and *Vamp8* expression compared to the WHTC group (Figure 7(b)).

WT mice were transplanted with the microbiome of WT or fat-1 mice and fed CTL or HF diet for 12 weeks. a: Colonic mRNA expressions of *Chop, Atf4*, and *Edem1 I* (*n* = 8 per group). b: Colonic mRNA expression of *Lc3, Ulk1, P62 and Vamp8* (*n* = 8 per group). Data are shown as mean ± SEM, and differences were analyzed by Tukey’s multiple comparison test. Bars assigned different superscript letters (a, b, c) are statistically different at *p* < 0.05. WT mice transplanted with microbiota of WT mice fed a CTL diet, WTW HFD: WT mice transplanted with microbiota of WT mice fed a HFD, WTF CTL: WT mice transplanted with microbiota of fat-1 mice fed a CTL diet, and WTF HFD: WT mice transplanted with microbiota of fat-1 mice fed a HFD. CHOP: C/EBP Homologous Protein. ATF4: Activating transcription factor 4. Edem1: ER degradation enhancer, mannosidase alpha-like 1. LC3: Microtubule-associated protein 1A/1B-light chain 3. ULK1: Unc-51 like autophagy activating kinase. P62: Sequestosome-1. VAMP8: vesicle-associated membrane protein; 8.

### Impact of HFD on microbiota analysis in WT mice transplanted with WT or fat-1 microbiome (Figure 8)

**Figure 8.**
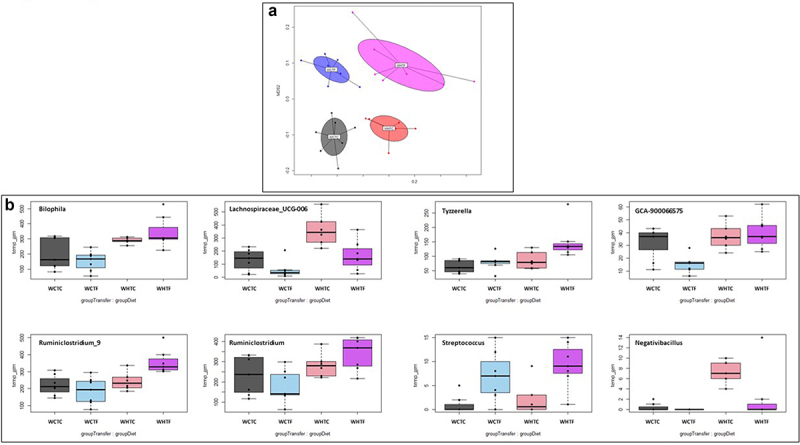
Impact of HFD on microbiota analysis in WT mice transplanted with WT or fat-1 microbiome.

Analysis of the fecal microbiota *via* 16S rDNA sequencing showed that the four communities were distinct after 12 weeks (Figure 8(a)). After correction for the false discovery rate, univariate analysis revealed that Streptococcus was the only genus that was significantly more abundant in the mice that received the fat-1 transplant, whereas Lachnospiraceae_UCG-006 and Tyzzerella were affected by the diet (see Supplemental Table S1). Transplantation of the fat-1 microbiome in HFD-fed WT mice significantly increased *Bilophila*, *Ruminiclostridium_9*, *Ruminiclostridium* and *Tyzzerella*. Moreover, *Negativibacillus* was significantly increased by HFD challenge in WHTC mice, but remained similar to controls when HFD-fed animals were transplanted with the fat-1 microbiome (Figure 8(b)). The diet also significantly impacted the Picrust2-predicted pathways, while the impact of the FMT was not significant. Indeed, the pathway of sucrose degradation IV (sucrose phosphorylase) decreased in the high-fat diet, whereas the superpathway of L-methionine biosynthesis (by sulfhydrylation) increased (see Supplemental Table S3).

WT mice were transplanted with the microbiome of WT or fat-1 mice and fed CTL or HF diet for 12 weeks. a: The NMDS representation of the microbiota of mice transferred with control donor or fat-1 donors allows discrimination of the four groups at week 12 based on the Bray-Curtis distance. The same color code as Figure 5 was used. The Stress in the NMDS representation was 16.9%. b: Major bacterial genera encountered in WT mice transplanted with the microbiome of WT or fat-1 mice and fed CTL or HF diet

### Impact of HFD on metabolites in the cecum of WT mice transplanted with WT or fat-1 microbiome (Figure 9)

**Figure 9.**
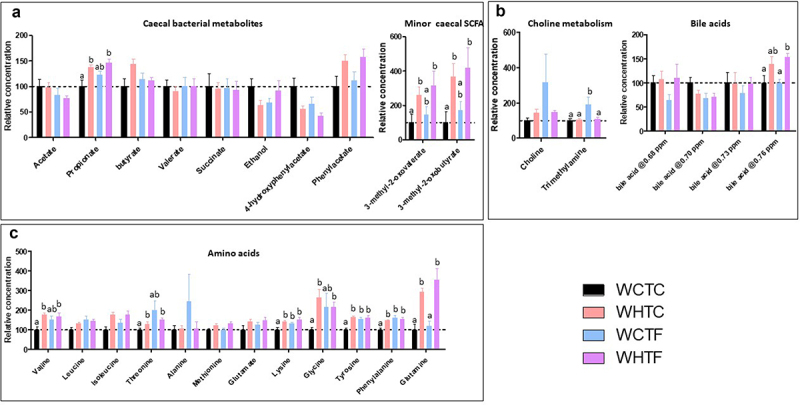
Impact of HFD on metabolites in the cecum of WT mice transplanted with WT or fat-1 microbiome.

The activity and composition of the gut microbiome are significantly affected by dietary sources.^41^ As the beneficial effects of the gut microbiota are mainly mediated by the secretion of various metabolites, we used nuclear magnetic resonance (NMR) to further characterize metabolites in the cecum. The levels of short-chain fatty acids (SCFA, propionate, 3-methyl-2-oxovalerate, and 3-methyl-2-oxobutyrate) and amino acids (valine, threonine, lysine, glycine, tyrosine, phenylalanine, and glutamine) were higher in mice fed an HFD, independent of the genotype of the microbiota donor. This increase in propionate was only observed in the feces of the WHTC animals (Figure 9(a)). Interestingly, the concentration of trimethylamine was increased in WCTF mice (Figure 9b), indicating an influence of the fat-1 microbiota on the production of this bacterial metabolite in mice fed the control diet, but not the HFD. The transfer of fat-1 microbiota also increased the levels of amino acids (lysine, tyrosine, and phenylalanine) in mice fed the control diet (WCTF) (Figure 9(c)).

WT mice were transplanted with the microbiome of WT or fat-1 mice and fed CTL or HF diet for 12 weeks. Metabolites were analyzed in the cecal content using NMR-based metabolomics. a: Relative concentrations of microbiota-derived metabolites. b: Relative concentrations of metabolites linked to choline metabolism and bile acids. For bile acids, the chemical shift of the peak used for quantification was indicated in ppm. c: Relative concentration of amino acids. Data are shown as mean ± SEM, and differences were analyzed by Tukey’s multiple comparison test. Bars assigned different superscript letters (a, b) are statistically different at *p* < 0.05.

## Discussion

High-fat diet is known to lead to gut microbiota dysbiosis, increased intestinal permeability, and consequent systemic inflammation.^42^ The mucus layer microenvironment remains a neglected niche in most microbiota studies; however, it is a key feature in the modulation of gut health. We then aimed to understand the molecular mechanisms by which the omega-3 PUFAs-modified microbiome modulates the colon mucus layer to decipher how it influences its structure and functionality.

When numerous studies revealed an association between obesity and a decrease in mucus layer thickness,^18,35,43^ which supports a mechanism of increased gut permeability (metabolic endotoxemia), in the current study, we demonstrated that 45% available energy as fat for a 11-week period significantly increases the thickness of the adherent mucus layer in the colon of WT animals. This increase in mucus thickness was further supported by the upregulated expression of *Muc*2, the major mucus-forming mucin within the colon. This result seems to indicate a direct effect of HFD on the expansion of the colonic mucus layer. Nevertheless, our results agree with a recent study by Liu and coworkers showing that in rats receiving HFD compared to controls, the colonic mucus layer was thicker and *Muc2* was overexpressed in association with low-grade colonic inflammation.^44^ These discrepancies might be due to the duration of the HFD (11 weeks in our study *versus* 22 in^18^ or the percentage of available energy as fat (45% compared to 60% in.^35^ In the literature, a thicker colon adherent mucus layer has been shown to be associated with greater protection of this part of the gut.^45,46^ However, our results clearly showed higher gut permeability in HFD-fed WT mice (Supplemental Figure S1C). Thus, we can imagine that the thicker mucus layer observed in the present study would exhibit an altered structure of MUC2 glycoproteins, leading to the observed functional modifications leading and loss of mucus viscoelastic properties and then consequently a loss of protective functions, as already observed in Crohn’s disease.^47^ These authors demonstrated that these effects were attributed to a reduction in the oligosaccharide chain length by 50% of MUC2.

As the thickness of the mucus layer is modulated by HF diet conditions, we thought it might be highly relevant to investigate the architecture of this viscous hydrogel, in which the main structural components are mucins and highly glycosylated and interconnected proteins. Consequently, we studied the complex and highly structured organization of the two colon mucus-gel layers by electron microscopy and found that HFD-fed WT mice exhibited a thicker, more heterogeneous, and looser mucin net-like structure of adherent mucus compared to CTL-fed animals, suggesting a more permeable barrier. Indeed, the presence of bacteria in the inner mucus layer of HFD-fed WT mice reinforced this hypothesis (Figure 2(a)). More specifically, we highlighted that MUC2 mucin-dependent mucus layer in colon is characterized by a dense well-organized stratified lamellar appearance in CTL-fed wild-type and CTL-fed fat-1 mice. Remarkably, this stratified mucus network organization has been preserved in HFD-fed fat-1 group (Figure 2(b)). These data suggest that it was formed by sheets of polymerized MUC2, as already evidenced by Johansson and coworkers in conventional mice.^48^ Such organization has been shown to be favored by the N-terminal trimers in the MUC2 polymers.^49^ The staggered layers of the MUC2 mucin act as a size-exclusion filter and does not normally allow bacteria to penetrate.^48,50^ In contrast, in HFD-fed WT mice electron microscopy evidenced a disorganized mucus layer architecture with no stratified lamellar appearance (Figure 2(b)). Our study clearly demonstrated, for the first time, that omega-3 tissue enrichment of HFD-fed fat-1 mice completely prevented mucin network disorganization and increase in the thickness of the mucus layer (which remained similar to WT and fat-1 control animals), maintained *Muc-2* and increased *Klf4* and *Tff3* gene expression. The altered mucus layer has been shown to allow bacteria to come closer to the intestinal epithelial cells and induce inflammation,^34,51–53^ leading to epithelial damage that allows bacterial components such as LPS to diffuse into the bloodstream and induce metabolic endotoxemia. The latter is associated with the onset of cardiometabolic disorders.^4^ Accordingly, protection against the development of obesity and associated metabolic disorders in fat-1 mice could most likely be accounted for by omega-3 PUFAs-preventive effects on architecture mucus layer disruption. We may then assume that, contrary to what is observed in HF-fed wild-type mice, the dense and well-organized stratified mucus layer observed in CTL-fed wild-type mice and fat-1 animals – whatever the diet – would form a specialized physical barrier that excludes the resident bacteria from a direct contact with the underlining epithelium and thus also the immune system which triggers inflammation and associated endoplasmic reticulum stress induction.

The primary translation product of MUC2 is rapidly covalently dimerized through the endoplasmic reticulum (ER) before undergoing O-glycosylation.^15^ ER stress has been evidenced to be the main cause of protein misfolding.^54^ Previous studies have shown that obese subjects display enhanced ER stress and an unfolded protein response (UPR) associated with intestinal barrier alteration,^18,55^ which is consistent with our results observed in HFD-fed WT animals. It is worth noting that markers of the colonic endoplasmic reticulum stress were alleviated in HFD-fed fat-1 mice, as evidenced by the downregulation of the protein expression of BIP, CHOP and p-eIF2α. A similar tendency was observed in *Chop*, *Edem1*, and *Grp78* gene expression. This suggests that omega-3 tissue enrichment, partly through its anti-inflammatory properties, maintains the expression of *Muc2* and preserves the mucus layer architecture by preventing ER stress in HFD-fed animals. These findings are in accordance with similar results obtained in HFD-fed mice treated with the anti-inflammatory IL (Interleukin)-22, which reduced ER/oxidative stress, low-grade intestinal inflammation, and improved integrity of the mucosal barrier.^18^ Moreover, the present data reinforce our previous results showing that omega-3 tissue enrichment protects mice against gut barrier dysfunction^32^ and strengthen our recently published *in vitro* data showing that EPA (eicosapentaenoic acid) and DHA (docosahexaenoic acid) were able to prevent the altered MUC2 production induced by palmitic acid (a major fatty acid found in HF diets), mainly by alleviating ER stress in well-differentiated human colonic LS174T goblet cells.^26^

MUC2 secretion and renewal of the colonic mucus layer are essential for its protective functions. Secretory autophagy has emerged as an alternative non-degradative mechanism for cellular trafficking and unconventional protein secretion.^36,37^ Goblet cells release stored mucin granules in response to autophagy and Ca^2+^-mobilizing agents. Indeed, ATG5-deficient mice are unable to release MUC2,^37^ and ATG7 knockout animals exhibit a diminished mucus layer and are more susceptible to dextran sulfate sodium-induced colitis.^56^ Unexpectedly, neither the diet nor the genotype-modified LC3 protein expression and the gene expression of a number of known autophagy markers. In addition, we found that mucin exocytosis decreased in HFD-fed WT mice, whereas a thicker mucus layer was observed. Indeed, it seems not to be in accordance with what has been recently observed in the literature indicating that omega-3 PUFAs or their derivatives improve metabolic syndrome features through autophagy improvement or autophagy-associated attenuation of ER stress in different tissues, such as skeletal muscle and liver .^57–59^ We can therefore assume that under our experimental conditions, the ER stress-associated alteration of the colonic mucus layer did not seem to involve the autophagy process.

Recently, it has become evident that mucus layer homeostasis is not entirely a host-controlled process. Among the factors influencing the mucus barrier, the gut microbiota strongly contributes to the properties of the colonic mucus layer and is required to fully mature this defense.^60,61^ We investigated the relative contribution of the microbiome to the protective effects of omega-3 PUFAs against alterations in the colonic mucus layer. For this, we transplanted the fat-1 cecal microbiome into WT mice fed an HFD for 12 weeks. Unlike we previously performed,^3^ in the present study, recipient WT mice were not treated with antibiotics prior to transferring the microbiome as they can selectively deplete components of the microbiota^62^ or even limit the establishment of an exogenous microbiota, as recently discussed.^63^ We evidenced a shift in microbiota composition from the basal state to the final microbiota (Supplemental Figure S6). Nonetheless, we confirmed here that WT mice fed an HFD and colonized with fat-1 microbiome exhibited significantly lower weight gain, improved glucose tolerance, and restored impermeability of the intestinal epithelium compared to WHTC mice. Moreover, extending our previous work,^3^ we focused in the present one on the integrity of the mucus layer and demonstrated for the first time that transplanting fat-1 microbiota also remarkably preserved the integrity of the colonic mucus layer in mice fed the HF diet. This was associated with the prevention of altered-*Muc2* and *Klf4* expression induced by HFD and the protection of HFD-fed WT mice against colonic ER stress. This suggests that the beneficial effects of omega-3 PUFAs in terms of preserving mucus layer integrity are a transmissible trait that accounts for most of the observed phenotypic differences between fat-1 and WT mice through ER stress improvement in HFD-fed mice. In order to get an idea of the omega-3 fatty acid levels in the gut luminal content of fat-1 *versus* wild-type mice, we have performed a total fatty acid composition in feces (Supplemental Figure S7). The results show that these fatty acid compositions reflect more or less the one of the diet in both genotype with an increase of very long-chain omega-3 fatty acids in the feces of fat-1 mice. Thus, we may assume that the effects of cecal microbiome transplantation might be due to microbiota changes and long-chain omega-3 fatty acids present in the gut luminal (as shown by feces analysis in Figure S7). The fact that the gut microbiome plays a key role in the intestinal mucus barrier has already been illustrated in a study using two genetically identical mouse colonies housed in different rooms in the same animal facility. The authors first demonstrated that the colonies presented two different gut microbiota compositions, and that one colony exhibited a colonic mucus layer impenetrable by bacteria or by beads with the size of bacteria, while the other one presented a highly penetrable mucus layer. Moreover, they showed that the gut microbiota is involved in the transmission of mucus properties have been transmitted after transplantation of the cecal microbiome to germ-free mice.^61^ Therefore, in the present study, we speculated that some bacteria and/or metabolites from fat-1 microbiota would have the ability to favorably modify the mucus layer toward a highly stratified non-penetrable mucus layer, whereas other phyla from WT mice would have opposite effects.

This assumption led us to perform gut microbiota composition and metabolomic analyses in the transplanted animals. Indeed, microbiota directly affects various biological processes of the host through the production or degradation of a multitude of compounds. Multiple bacteria can modulate metabolic reactions, resulting in combinatorial metabolism of substrates by the microbiome and host, exemplified by the production of bile acids, choline, or SCFA. Our data showed that the transplantation of the fat-1 microbiome into HFD-fed WT mice significantly increased *Bilophila*, *Ruminiclostridium_9*, *Ruminiclostridium*. We believe that these taxa may play important roles in the omega-3 PUFAs-modified microbiota in colon homeostasis in HFD-fed mice. In line with this, Gao and coworkers et al. recently showed in HFD mice supplemented with α-linolenic acid (18:3 omega-3) that *Bilophila* was positively correlated with *Occludin* and *MUC2* in the ileum and colon, respectively, and negatively correlated with *TLR4* in the colon.^64^ In addition, *Bilophila* is widely regarded as a potentially harmful genus or a conditional pathogen, as *Bilophila wadsworthia* strain aggravates HFD-induced metabolic dysfunctions in mice.^65^ However, the results of previous studies have been contradictory. Previous studies have reported that the decrease in *Bilophila* was related to the occurrence of the disease, as a decreased abundance of the genus *Bilophila* was evident at the inflamed sites of patients with ulcerative colitis compared with the corresponding sites in non-IBD controls.^66^ Consequently, we believe that the function of *Bilophila* might be complex and conditioned by omega-3 PUFAs, which is worthy of further study. *Ruminiclostridium_9* (a butyrate-producing bacterial member from the Ruminococcaceae family) has been implicated in positive health states. More specifically, HFD consumption in a rat model decreased the relative abundance of *Ruminiclostridium*, whereas a prebiotic diet recovered it.^67^ In addition, it has been shown that a polyphenol-rich fruit extract ameliorates glycolipid metabolism in obese mice and type-2 diabetic rats, accompanied by an increase in the number of *Ruminiclostridium_9*.^68^ As a SCFA producer, *Ruminiclostridium_9* is associated with the release of inflammatory and cytotoxic factors from the gut to maintain a stable intestinal microecology.^69^ Thus, we may assume that the genera *Bilophila* and *Ruminiclostridium_9* play crucial roles in the improvement of intestinal mucosal barrier integrity. Nevertheless, it has also been pointed out that administering HFD increased *Ruminiclostridium_9*, whereas administering HFD and resveratrol (the most well-known anti-inflammatory polyphenolic stilbenoid found in gapes, mulberries, and multiple other plants) reduced the content of *Ruminiclostridium_9*. In addition, the authors compared the microbiota of mice fed a normal diet with those fed a normal diet and resveratrol and found an increase in *Ruminiclostridium_9*.^70^ It is speculated that different diet compositions will cause different microbiota, and the host’s obesity or leanness may also cause differences in the composition of the microbiota. Interestingly, we found that *Negativibacillus*, known to be correlated with metabolic endotoxemia since it is positively related to LPS, TNF (Tumor Necrosis Factor)-α, IL-6, and Il-1β in serum,^64^ was significantly increased by HFD challenge in WHTC mice but remained similar to controls when HFD-fed animals were transplanted with the fat-1 microbiome. An altered microbial community can result in differences in metabolite production, including SCFA, which have been described to modulate mucus expression.^71^ We observed that the levels of SCFA (propionate, 3-methyl-2-oxovalerate, and 3-methyl-2-oxobutyrate) and amino acids (valine, threonine, lysine, glycine, tyrosine, phenylalanine, and glutamine) were higher in mice fed a HFD, independent of the genotype of the microbiota donor. We may conclude that microbiota-derived metabolites do not seem to be the major drivers for preventing mucus defects under our experimental conditions. The changes in bacterial composition observed in the current study suggest that omega-3 PUFAs beneficially modulate the functional groups within the gut microbiota of HFD-fed mice, indicating a likely mechanism by which these treatments relieve intestinal alterations and restore intestinal barrier structure.

Different hypothesis can explain the role of omega-3 PUFAs on microbiota modulation. We previously showed that beside significant differences in cecal microbiota between WT and fat-1 mice, these latter display significantly greater bacterial phylogenic diversity than do WT mice when both are fed the HF diet. Cells of the gastrointestinal tract – including the lower part of the intestine where the bacteria are the most abundant – are highly enriched in omega-3 fatty acids due to the expression of the fat-1 gene.^3^ So, because the intestinal epithelium is continuously renewed, we may speculate that a “cellular” source of long-chain omega-3 fatty acids brought to the microbiota from the intestinal cell shedding would explain how the microbiota would also be “fed” by omega-3 fatty acids and would consequently have a positive effect. Moreover, it has already been evidenced that omega-3 PUFAs share the important immune system activation/inhibition pathway with gut microbes modulating pro-inflammatory profiles.^72^ Several types of fatty acids provide an antimicrobial activity which occurs after the complete enzymatic hydrolysis of lipids by the gut microbiota in the lower gastrointestinal tract.^73^ This antimicrobial activity depends on the length of their carbon chain and on the presence, number, position, and orientation of double bonds; omega-3 PUFAs being fatty acids with the longest and most unsaturated chains. Elsewhere, studies on mice-transplanted feces showed that the omega-3 PUFAs can modify the microbiota through the production and secretion of intestinal alkaline phosphatase (IAP), leading to a reduction of the number of LPS-producing bacteria, thus reducing metabolic endotoxemia.^74^

One of the key remaining question is : what are the mechanisms underlying the protective effect of fat-1-altered gut microbiota on mucus defects? Among the possible answers, the pattern of expression of glycosyltransferases might be considered. If microbes can use mucus as a prominent energy source, they could also induce changes in mucus rheological properties through hydrolysis of O-glycans units in mucus glycoproteins such as MUC2.

Indeed, both the presence and the number of specific bacteria shape the glycan profile of the mucus and are directly associated with numerous glycosyltransferases whose levels are modified in the presence of the gut microbiota.^13,75^ For example, it has been observed that some bacteria are able to induce the expression of host fucosyltransferases, which add L-fucose at the α − 1,2 position and sialyltransferases.^13^ Furthermore, host bacterial communities are able to affect both MUC2 glycosylation and the glycosylation of transmembrane mucins.^76^ It has been shown that feeding a Western-style diet leads to increased penetrability of the colonic mucus layer which could be due to increased degradation of bacterial glycan, but could also be caused by a host defect leading to decreased production, secretion or assembly of the MUC2 mucin.^77^ On the other hand, our results show that omega3 tissue enrichment of fat-1 mice increases specific bacteria community-like *Rumminococcus* that produces mucus-degrading enzymes like α-galactosidases and α-N-acetylgalactosaminidases,^78^ which could exert a beneficial effect on the mucus layer integrity.

Overall, such changes in mucus resident bacteria can modify the syntrophic, symbiotic and mutualistic interaction of microbes wthin the mucus layer creating an environment favoring microbial community selection and defining physical properties of the mucus layer favorable to an alleviation of gut permeability and thus obesity-related metabolic disorders.

Generally, male mice are used in diet-induced obesity (DIO) studies as female mice are less sensitive to diet-induced obesity and less affected by associated metabolic disorders. Indeed, Orsso and coworkers^79^ stated that male and female present specific differences in body composition, adipose tissue distribution, and metabolism. Sex hormones have been shown to influence eating behavior, in the so-called “homeostatic” control of energy intake as well as “the hedonic” control of food intake^80^ and disturbances linked to obesity.^81^ Physiologically, estrogens influence food intake by both central (i.e., hypothalamic circuits) and peripheral signals. Indeed, studies in mice have demonstrated that reduced estrogen levels may induce hyperphagia.^80^ Conversely, gonadectomized males reduce food intake.^82^ When the influence of the microbiota is studied, several studies have demonstrated that gender influences gut microbiota composition^83,84^ and it has also been shown that under sufficiently extreme conditions revealed by well-controlled murine environments, diet can have divergent effects on the gut microbiome based on sex. For example, Bolnick and coworkers demonstrated that in male mice, *Lactobacillus*, *Alistipes*, *Lachnospiraceae*, and *Clostridium* were more abundant on a high-fat rather than a chow diet, while in females, these genera were less abundant on a high-fat diet.^85^

Considering the limitations of the present study, we did not compare our strategy with other models (germ-free for instance). Nevertheless, it has already been shown the possibility to transfer a lean phenotype in both germ-free mice^86^ and the present model.^87^ Currently, there is no consensus on animal models of intestinal microbiota transplantation and researchers should choose the model based on the disease studied (*i.e*. obesity). All available models, including ours, have their own advantages and disadvantages. It is important to be aware of them and choose the best model depending on the goal of the study. Most studies on intestinal microbiota transplanted mice have focused specifically on bacteria shared by the intestinal microbiota of donor and recipient mice, including ours. Although this is an important feature, intestinal microbiota transplantation includes transfer of more than bacteria. Viruses, archaea, and fungi are also components of feces and may affect recipient biology,^88^ as it was shown that fungi are involved in the pathophysiology of alcoholic liver disease and cirrhosis.^89,90^ Thus, the effects of these different components can also be studied. In addition, we did not compare the transgenic production of omega-3 PUFAs to a dietary supplementation of these fatty acids on mechanisms underlying protection against obesity and metabolic disorders. Nevertheless, it has recently been shown that both models reveal distinct but overlapping mechanisms underlying metabolic disorders.^91^ Moreover, it has been previously evidenced that the two models also similarly affected the gut microbiota composition. Indeed, separate studies reported that fat-1 mediated omega-3 PUFAs production^3,74^ as well as omega-3 PUFAs supplementation^92,93^ are able to modulate bacterial populations. Disturbances in DIO studies being known to be modulated by female sex homones, only male mice have been used in our studies. Nevertheless, it would be worthwile to compare data in both genders in view to understand the influence of estrogens on gut microbiota and lean phenotype transmission.

Given the link between ER stress, mucus production, and inflammation, many researchers and clinicians have begun to focus on reducing ER stress and promoting a highly stratified nonpenetrable mucus layer as a potential therapeutic target for cardiometabolic disorders. Our study highlights the novel role of omega-3 PUFAs in alleviating ER stress, promoting mucin-modulating properties, and minimizing inflammation. Based on these findings, we believe that omega-3 PUFAs could serve as next-generation mucin-building prebiotics for intestinal diseases associated with ER stress and disrupted mucus, such as dietary obesity.

## Materials and methods

### Animals

Wild-type (WT) and fat-1 transgenic mice (FAT1)^33^ were housed in a conventional clean facility on a 12-h light/dark cycle and fed *ad libitum* with a 10% safflower oil diet (Ssniff), as unique source of lipids, in order to exacerbate omega-3 tissue enrichment in fat-1 mice. All procedures followed institutional guidelines for the use and care of laboratory animals and were approved by the Ethics Committee of the University of Burgundy (approval number APAFIS#15830).

### Diet-induced obesity experiment

Twenty WT and 20 fat-1 mice (12–16-week-old) were housed in individual cages. Animals of each genotype were divided into two subgroups of 10 animals fed *ad libitum* a control (CTL) diet (EF D12450B; Ssniff) or a high-fat diet (HFD) (EF D12451; Ssniff) for 11 weeks. Both CTL and HFD contained 20% protein, 10 and 45% fat, and 70 and 35% carbohydrates, respectively. The omega-6-to-omega-3 ratios in the diets were 8.36 (CTL) and 10.88 (HFD).

In order to block autophagy flux, mice were treated 4 h before sacrifice with leupeptin (40 mg/kg by intraperitoneal injection) to inhibit hydrolases and proteases and then monitor the accumulation of autophagosomes and increase in LC3-II proteins. The groups were labeled as follows: WT fed the CTL diet (WT CTL), WT fed the HFD (WT HFD), fat-1 fed the CTL diet (FAT1 CTL), and fat-1 fed the HFD (FAT1 HFD).

### Caecal microbiota transplantation experiment

Twelve to sixteen-week-old male donor mice, WT or fat-1, were killed by cervical dislocation. Caecum was ligated, quickly removed from the animal, and transferred to an anaerobic chamber in which cecal content was resuspended in 1 ml of Ringer’s solution (9 g/L NaCl, 0.4 g/L KCl, CaCl_2_ 0.25 g/L, L-Cysteine hydrochloride 0.05%). For each transplantation, the cecal contents (homogenized in 1 ml of Ringer’s solution per animal) of seven wild-type or seven fat-1 donors were collected and homogenized together in an anaerobic chamber which allowed us to get close to 10 ml of WT and 10 ml of fat-1 cecal content homogenate. These homogenates were sufficient for all instillations: indeed, 200 µl of the WT cecal content homogenate were administered by gavage to CTL or HFD-fed WT recipient mice (8 per group) and 200 µl of the fat-1 cecal content homogenate were administered by gavage to CTL or HFD-fed WT recipient mice (8 per group). Thirty-two WT mice (12–16 weeks old), housed in individual filter-covered cages, were divided into four subgroups of eight animals and fed *ad libitum* the CTL or HFD *ad libitum* for 12 weeks. Two subgroups received cecal microbiota from male FAT1 mice (12–16 weeks old) donors, while the other two subgroups received cecal microbiota from WT littermate control (12–16 weeks old) donors. Animals were transplanted once a week during the first three weeks and thereafter once a week every two weeks until they were sacrificed. These four subgroups were labeled as follows: WT transplanted with WT microbiota fed a CTL diet (WCTC), WT transplanted with WT microbiota fed an HFD (WHTC), WT transplanted with fat-1 microbiota fed a CTL diet (WCTF), and WT transplanted with fat-1 microbiota fed an HFD (WHTF). A food consumption of animals has been followed on weeks 6 and 8.

### Insulin and oral glucose tolerance tests and intestinal permeability assay

The Insulin Tolerance Test (ITT) was performed at week 9. Briefly, mice were fasted for 6 h and received an oral load (2 g/kg) of a D-glucose solution (20% w/v) followed by an intraperitoneal injection of insulin (0.5 UI/kg; Actrapid; Novo Nordisk, Paris, France). Glycemia was measured at 0, 15, 30, 60, and 90 min after glucose loading directly into blood samples from the tail vein using a One Touch Verio Glucometer (LifeScan).

Oral Glucose Tolerance Test (OGTT) was performed at week 11 as previously described.^94^

At week 12, intestinal permeability was assessed by force-feeding mice with fluorescein isothiocyanate (FITC)-dextran 4 kDa (600 mg/kg body weight, 120 mg/mL), collecting blood from the retro-orbital vein 4 h after gavage, and measuring plasma fluorescence of the FITC-Dextran 4 kDa flux. At the end of the study, the 6 h-fasted mice were killed by lethal intracardiac blood puncture, and the colon was collected and fixed for imaging or snap-frozen in liquid nitrogen, and stored at −80°C until further analysis.

### Determination of the thickness of the colonic mucus layer, density and size of mucin granules

Samples were prepared and analyzed at the CellImaP core facility (INSERM U1231, Dijon, France). Distal colons containing a feces were collected and immediately placed in Carnoy’s fixative solution (60% methanol, 30% chloroform, and 10% acetic acid) for 8 h and then transferred to 100% ethanol for 24 h. They were then embedded in paraffin and 5-µm-thick sections (two different levels per block) were deposited onto Superfrost Plus slides. After deparaffinization and rehydration steps, samples were stained in a 0.2% Alcian blue solution, rinsed in tap water, and counterstained with Harris hematoxylin.

Slides were imaged on an Axioscope A1 microscope (Zeiss France S.A.S) equipped with a Jenoptic Gryphax camera and using a 40X or 20X objective to visualize, respectively, secreted mucins (measurement of thickness of colonic mucus layer) and mucins in Goblet cells. The measurements were evaluated manually using ImageJ software and reported in an Excel sheet. For the measurement of mucus layer thickness, a minimum of eight images per mouse tissue was acquired and a minimum of eight measurements per image was performed. For mucins in Goblet cells, two mice per group and seven measurements per animal were performed. All images were taken under the same exposure conditions without autoscaling.

### Transmission electron microscopy

Colon segments containing feces (1 cm) were stored in a cold fixative solution containing 2.5% (v/v) glutaraldehyde in 0.1 M sodium phosphate buffer, pH 7.2. After 2 h of fixation at 4°C (ice) under vacuum degassing, feces-loaded colon specimens were rinsed thrice with the same phosphate buffer. The specimens were post-fixed for 1 h at 4°C in 1% (w/v) osmium tetroxide in 0.1 M sodium phosphate buffer, before being dehydrated through a graded ethanol series and propylene oxide, and embedded in Epon (Embed812). Ultrathin sections (90 nm) were cut using an ultramicrotome (model Reichert Ultracut E, Leica) equipped with a diamond knife and deposited on 300-mesh palladium copper grids. The embedded sections were stained with uranylesslead citrate and examined using an HT7800 electron microscope (Hitachi) operating at 100 kV and equipped with two advanced microscopy technique (AMT) cameras (Woburn).

### BiP (binding immunoglobulin protein) immunolabelling

Distal colons were formalin-fixed (paraformaldehyde, PFA 4%) and embedded in paraffin. Paraffin blocks were sectioned (5-µm-thick slices, two different levels per block), and colon slices were deposited onto Superfrost Plus slides. BiP (rabbit mAb C50B12, Cell Signaling Technology, two slides per block at two different levels) immunohistochemistry was performed as previously described.^95^

### LC3 and Vamp8 immunofluorecence

Mouse colons were fixed without flushing the luminal content with formalin, embedded in paraffin, and sectioned at 5 µm. After deparaffinization and serial rehydration steps, sections were subjected to antigen retrieval in citrate buffer pH 6.0 at 90°C for 30 min and blocked with 8% bovine serum albumin in PBS-Tween (0.1%). Slides were incubated overnight in a humidified chamber at 4°C with primary antibodies for staining LC3 (Sigma Aldrich #L7543, 1:200 diluted in 3% bovine serum albumin) and Vamp8 (Biotechne #AF5354, 1/30 diluted in 3% bovine serum albumin). The following day, slides were washed with PBS containing 0.1% Tween and incubated at RT for 2 h with fluorescent secondary antibodies (1:250 dilution). The LC3 primary antibody was detected using donkey anti-rabbit Alexa Fluor 488 antibody (Invitrogen #A-11058), and Vamp 8 primary antibody using donkey anti-goat Alexa Fluor 594 (Invitrogen #A-21206). Prolong Diamond Antifade Mountain with DAPI (Invitrogen #P36966) was used as a counterstain. Images were obtained using an immunofluorescence microscope (Zeiss). The analysis of antibody fluorescence intensity was performed using the ImageJ software (40X objective magnification). Fluorescence intensity was measured in several separate images and multiple regions of interest (ROIs) within an image, as indicated in the figure legends. The correlation was calculated in ImageJ using the Pearson correlation reported for the surface area of multiple ROIs per section. Each dot on the graph represents an ROI.

### c PCR

Total colon RNA extraction and quantitative PCR (qPCR) were performed as previously described.^3^ Total RNAs was extracted according to the manufacturer’s instructions (Qiagen #7406). One microgram of total RNA was reverse-transcribed using the High Capacity DNA Reverse Transcription kit (Applied Biosystems #4368813). cDNA were quantified by StepOne Plus real-time qPCR system (Applied Biosystems) via Power Syber Green PCR Master mix (Applied Biosystems 4,368,708) and the following primers (Eurogentec Company): MUC2 (F-5'CCT GAA GAC TGT CGT GCT GT3', *R*-5'GGG TAG GGT CAC CTC CAT CT3'), MUC4 (F-5'ACC TGG GGT GAC TTC CAT T3', *R*-5'CGT TGG TGT AGG CAT CGT TC3'), KLF4 (F-5'AGC CAC CCA CAC TTG TGA CTA TG3', *R*-5'AGT GGT AAG GTT TCT CGC CTG TG3'), CHOP (F-5'GGT GGC AGC GAC AGA GCC AG3', *R*-5'TGC CAT GAC TGC ACG TGG ACC3'), Edem1 (F-5'AAG CCT GCA ATG AAG GAG AA3', *R*-5'CTA TCA GCA CCT GCA GTC CA3'), Grp78 (F-5'GCG TGT GTG TGA GAC CAG AAC CG3', *R*-5'CAT CAT GCC GGC GCT GAG GA3'), ULK1 (F-5' AAG TTC GAG TTC TCT CGC AAG-3', *R*-5' CGA TGT TTT GCT TTA GTT CC-3'), LC3B (F-5' CCC ACC AAG ATC CCA GTG AT-3', *R*-5' CCA GGA TGG TCT TGT CCA-3'), ATF4 (F-5' GGA CGA TCT CTA ACG CCA CA-3', *R*-5'CTT GTC GCT GGA GAA CCC AT-3'), Tff3 (F-5' CCT GGT TGC TGCG GTC CTC TGG-3', *R*-5' GTC TCC TGC AGA CGT TTG AAG C-3'), P62 (F-5' GAG GCA CCC CGA AAC ATG G-3', *R*-5'ACT TAT AGC GAG TTC CCA CCA-3'), Vamp8 (F-5'GGA CCA CCT CCG AAA CAA GA-3', *R*-5' AGG GCT CCT CTT GGC ACA TA-3') and 18S (F-5' GTG TGG GGA GTG AAT GGTC-3', *R*-5' GCG AGA CAG TCA AAC CAC-3'). All reactions were performed in 96-wells plates with melting curves to ensure primer specificity. Finally, the relative mRNA levels were examined using the *ΔΔCT* method with the 18S housekeeping gene, generating fold changes for each gene.

### Western blot analysis

Colon tissues were homogenized in 300 µL RIPA lysis buffer containing a protease inhibitor cocktail (Sigma-Aldrich #P8340), centrifuged at 12,000 g for 15 min, and the supernatants were collected. The supernatant protein concentrations were quantified using a BCA protein assay kit (Thermo Fisher Scientific #23227). Proteins (40 µg/lane) were electrophoresed on 15% SDS gel and transferred to nitrocellulose membranes. Membranes were then blocked with 5% bovine serum albumin (BSA) at least 1 h at RT and the primary antibodies were incubated overnight at 4°C against β-actin (1:5000, Santa Cruz Biotechnology), LC3-II, and p62 (1:1000, Sigma-Aldrich and BD transduction Laboratories, respectively), CHOP (L63F7) (1:1000, Cell Signaling, #2895), KLF4 (1:1000, Cell Signaling, #4038), eIF2α (1:1000, Cell Signaling, #9722) and Phospho-eIF2α (Ser51) (1:1000, Cell Signaling, #9721). The membranes were incubated with HRP-linked anti-rabbit antibody for LC3, KLF4, eIF2α, Phospho-eIF2α and anti-mouse IgG secondary antibody for, P62 and CHOP, (1:10000, Cell Signaling Technology) for 1 h at RT. Protein bands were visualized using a chemiluminescence detection kit (Clarity^TM^ Western ECL Substrat #170–5061). All images were captured using Bio-Rad Chemidoc and analyzed using Image Lab software (Bio-Rad Universal Hood III). The expression levels of p62, CHOP and KLF4 were normalized to that of β-actin.

### Plasma LPS measurement

Plasma LPS measurements were assayed by direct quantification of 3-β-hydroxymyristic acid (3β-OH C14:0, 3HM, the most abundant hydroxylated fatty acid of the lipid A moiety of most LPS molecules) using gas high-performance liquid chromatography/tandem mass spectrometry (HPLC/MS/MS), as previously described.^96^

### Caecal microbiota analysis

Microbial profiling was performed as described previously.^97^ Briefly, microbial DNA was extracted using the Quick-DNA™ Fecal Microbe Miniprep Kit™ (Zymo Research #D4300) and a 15 min bead-beating step at 30 Hz was applied. The V3-V4 region was then amplified from diluted genomic DNA using the primers F343 (CTTTCCCTACACGACGCTCTTCCGA-TCTTACGGRAGGCAGCAG) and R784 (GGA-GTTCAGACGTGTGCTCTTCCGATCTTACCAGGGTATCTAATCCT) for 30 amplification cycles at an annealing temperature of 65°C. This V3-V4 region has proven useful in studying the variability of the pig microbiota in previous studies.^98,99^ The ends of each read overlap and are stitched together. In a single run, it generated extremely high-quality full-length reads of the full V3 and V4 regions. The Flash software v1.2.6^100^ was used to assemble each pair-end sequence, with at least a 10-bp overlap between the forward and reverse sequences, allowing 10% mismatch. Single multiplexing was performed using an in-house 6bp index, which was added to R784 during a second PCR with 12 cycles using a forward primer (AATGATACGGCGACCACCGAGATCTACACTCTTTCCCTACACGAC) and reverse primer (CAAGCAGAAGACGGCATACGAGAT-index-GTGACTGGAGTTCAGACGTGT). The resulting PCR products were purified and loaded onto an Illumina MiSeq cartridge, following the manufacturer’s instructions. Run quality was internally checked using PhiX, and each pair-end sequence was assigned to its sample using the integrated index with bcl2fastq Illumina software. The sequences were submitted to the Short-Read Archive under the accession number PRJNA946706. Filtering and trimming of high-quality sequences were applied to the reads using the DADA2 package in R software^101^ with the following parameters: maxN = 0, maxEE = 2, truncQ = 2, trimleft = 17. Chimeras were removed using the consensus method to obtain the final OTU abundance table. Taxonomic annotation using the assignTaxonomy function of dada2 with the Silva Dataset v132.^102^ The abundance matrix was rarefied to 9637 sequences per sample.

### Nuclear magnetic resonance-based metabolomics

Nuclear magnetic resonance (NMR) was used to analyze the metabolome in the cecal content, as described previously.^103^ Metabolites were identified by comparison with the spectra of the pure compounds. A representative spectrum is shown in Figure S5. The correspondence between numbers and metabolites is presented in Table 1. For each metabolite, the area under the curve of a 0.01 ppm segment corresponding to one peak was used for quantification (Table 1). Relative concentrations are expressed as a percentage of the mean value in the WCTC group.

**Table 1.** Identification of metabolites in cecal content NMR spectra.

	Metabolites	δ^1^H (ppm)
1	Bile acids (mixed)	0.68* (s), 0.70* (s), 0.73* (s), 0.76* (s)
2	Valerate	0.89 (t), 1.30* (m), 1.53 (m)
3	Butyrate	0.90* (t), 1.56 (m), 2.16 (t)
4	Leucine	0.97* (d)
5	Valine	1.00* (d), 1.05 (d), 3.62 (d)
6	Isoleucine	1.01* (d)
7	Propionate	1.06* (t), 2.19 (q)
8	3-methyl-2-oxovalerate	1.10* (d)
9	3-methyl-2-oxobutyrate	1.13* (d)
10	Ethanol	1.19* (t), 3.66 (q)
11	Threonine	1.34* (d), 3.60 (d), 4.26 (m)
12	Alanine	1.49* (d)
13	Acetate	1.92* (s)
14	Methionine	2.14* (s)
15	Glutamate	2.35* (m)
16	Succinate	2.41* (s)
17	Glutamine	2.46* (m)
18	Trimethylamine	2.88* (s)
19	Lysine	3.03* (t)
20	Choline	3.21* (s)
21	Glucose	3.26 (t), 3.39–3.56 (multiple signals), 3.70–3.75 (multiple signals), 3.81–3.92 (multiple signals), 5.24* (d)
22	Methanol	3.36* (s)
23	4-hydroxyphenylacetate	3.45 (s), 6.87* (d), 7.17 (d)
24	Glycine	3.57* (s)
25	Xylose	5.21* (d)
26	Galactose	5.27* (d)
27	Uracil	5.81* (d), 7.55 (d)
28	Tyrosine	6.91* (d), 7.20 (d)
29	Phenylacetate	7.30* (d)
30	Phenylalanine	7.34 (d), 7.38 (d), 7.43* (t)
31	Hypoxanthine	8.22* (d)
32	Formate	8.46* (s)

The metabolites identified in the NMR spectra of the ceacal content are presented. These numbers are reported in the representative spectra in Supplemental Figure S3. s, singlet; d, doublet; t, triplet; q, quintuplet; m, multiplet; *, peak used for quantification.

### Statistical analysis

Data are expressed as the mean ± SEM for each group. Because the limited sample sizes prevented proper verification of parametric test assumptions, groups were compared using Two-way ANOVA Tukey’s multiple comparison test. All analyses were performed using the GraphPad Prism 8.3.0 software (GraphPad Software Inc.). Microbiota data were analyzed as a community *via* NMDS based on the Bray-Curtis distance, and the impact of the transfer and the diet was analyzed by a linear model of the Y~Diet x Transfer_group, which was then corrected for the false discovery rate using the Benjamini Hochberg procedure. Metabolomic data were log10 transformed before analysis with a linear model including the effect of diet, donor of microbiota, and their interaction in R (4.2.0). Means were compared pairwise using the Tukey’s method.

## Supplementary Material

Supplemental Material

## Data Availability

The Illumina MiSeq cecal microbiota analysis sequences were submitted to the Short-Read Archive with accession number PRJNA946706.
