# Supplementary figures and images for: The transplantation of the gut microbiome of fat-1 mice protects against colonic mucus layer disruption and endoplasmic reticulum stress induced by high fat diet
Source: Gut Microbes. 2024 May 26;16(1):2356270. doi: 10.1080/19490976.2024.2356270 (PMC11135845; doi:10.1080/19490976.2024.2356270)

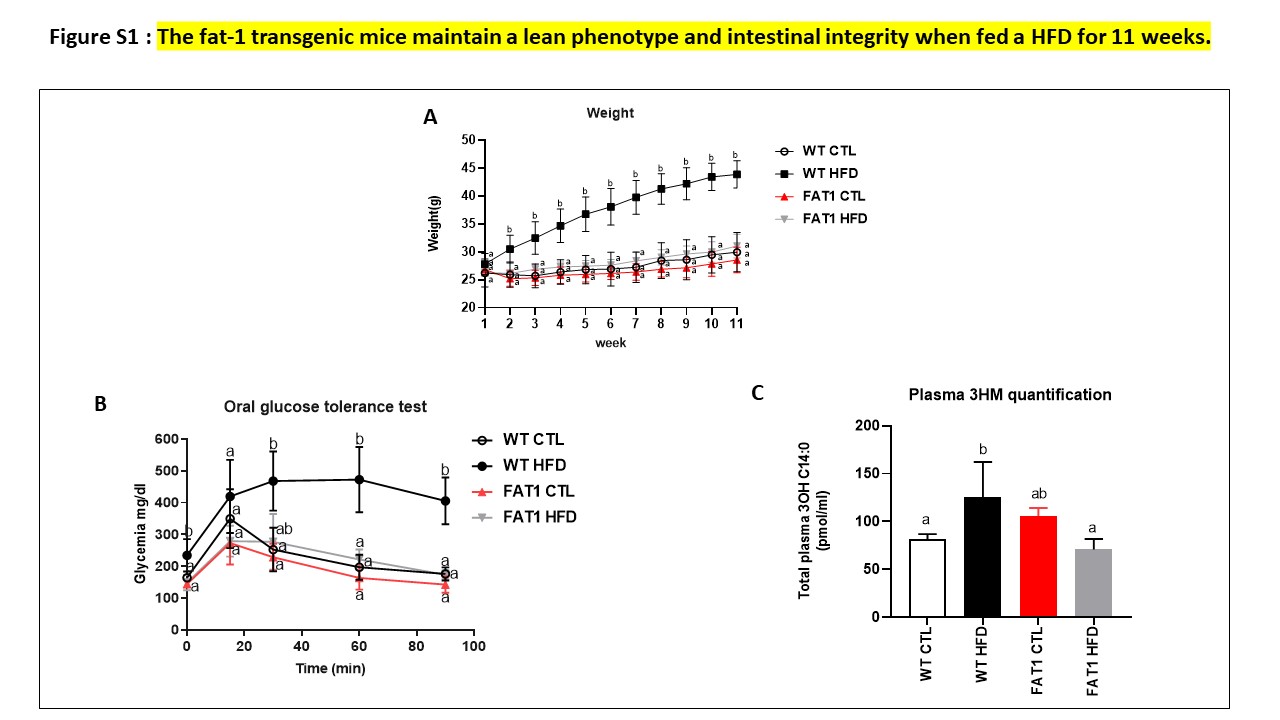

Supplement: Supplemental Material [file KGMI_A_2356270_SM0177.zip › FIGURE S1.jpg]

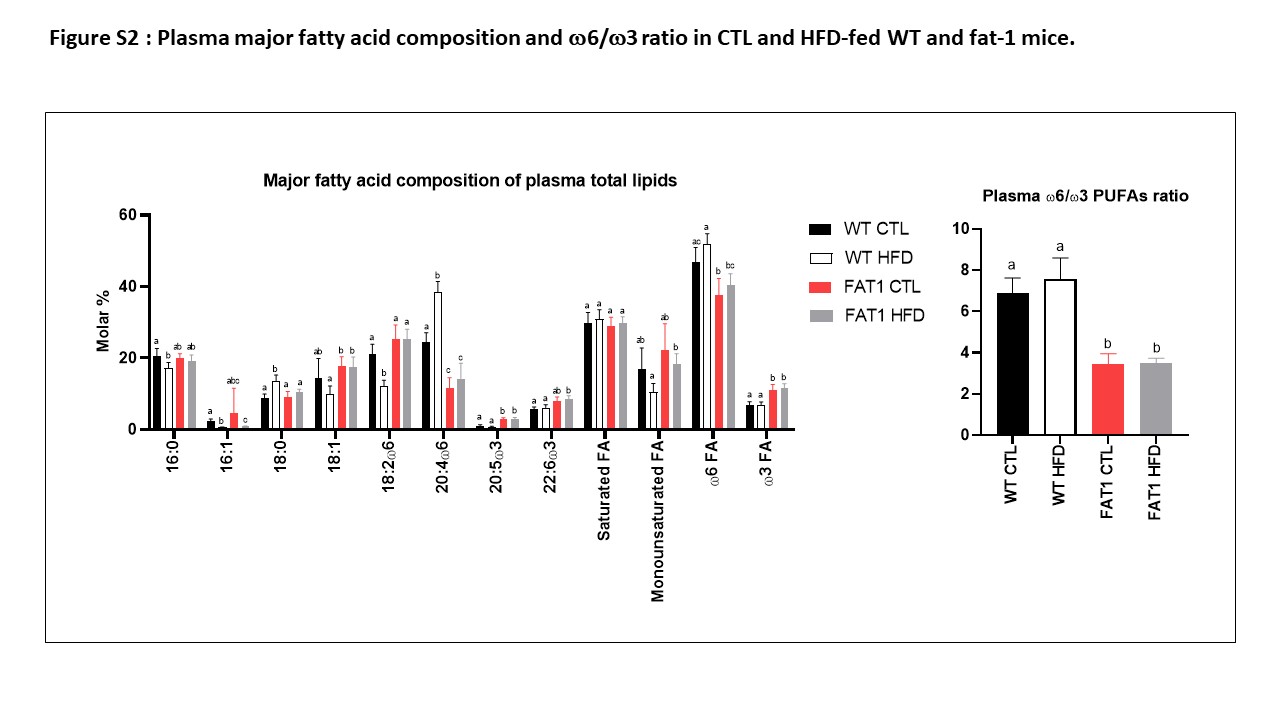

Supplement: Supplemental Material [file KGMI_A_2356270_SM0177.zip › FIGURE S2.jpg]

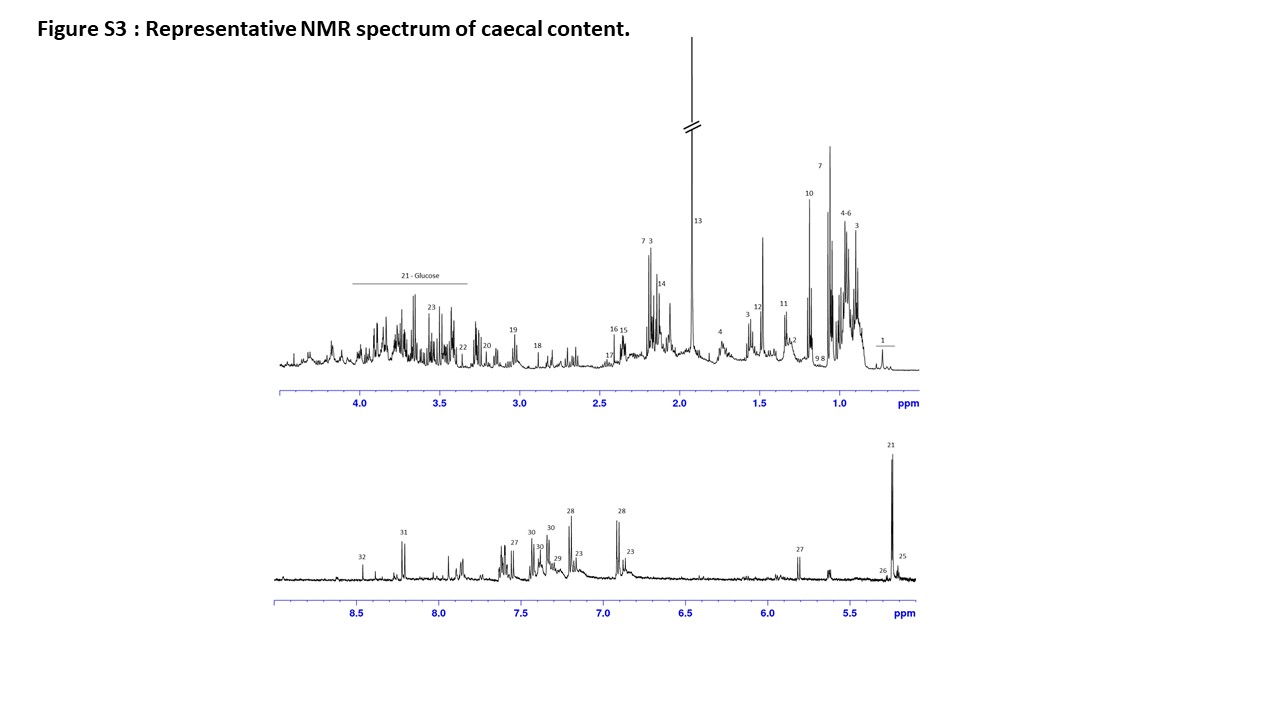

Supplement: Supplemental Material [file KGMI_A_2356270_SM0177.zip › FIGURE S3.jpg]

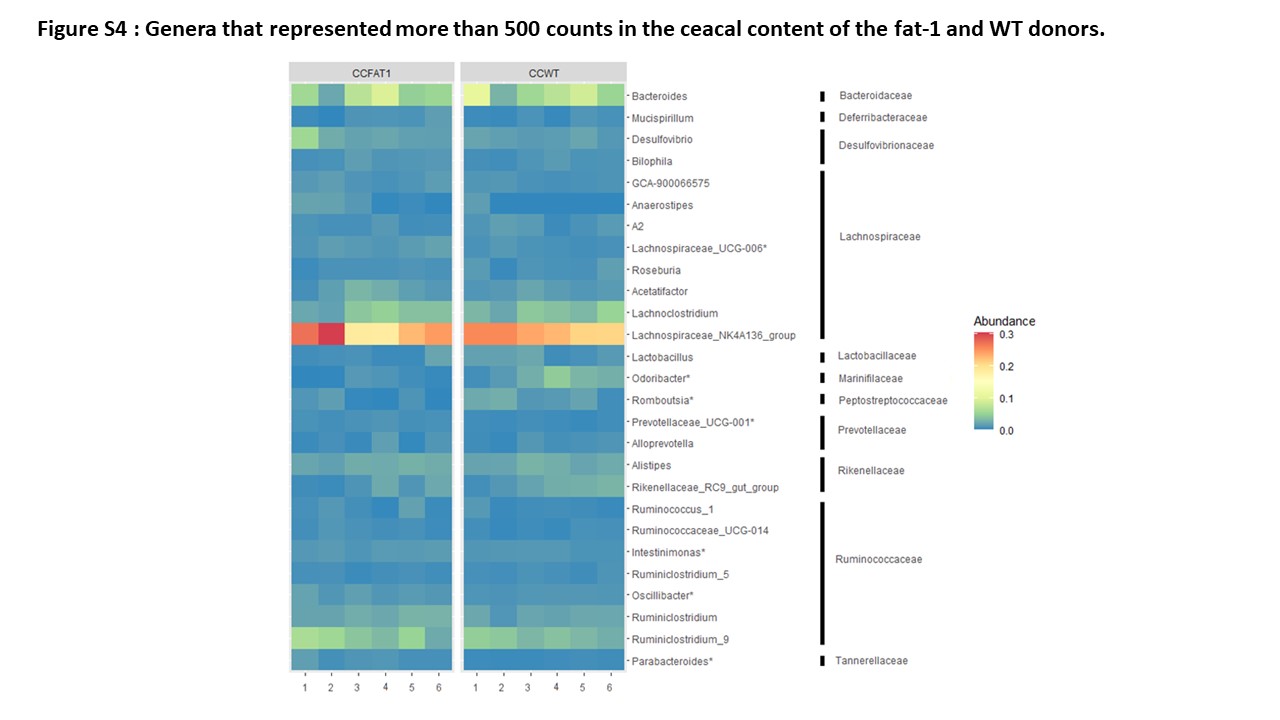

Supplement: Supplemental Material [file KGMI_A_2356270_SM0177.zip › FIGURE S4.jpg]

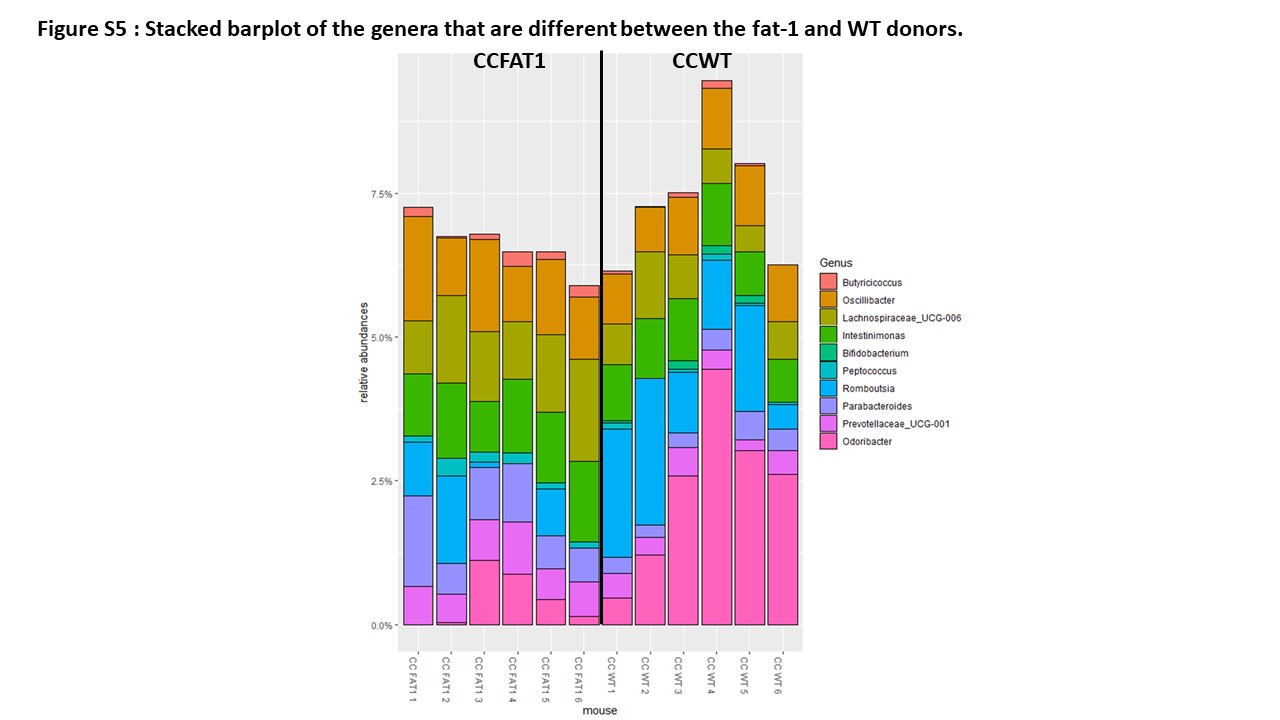

Supplement: Supplemental Material [file KGMI_A_2356270_SM0177.zip › FIGURE S5.jpg]

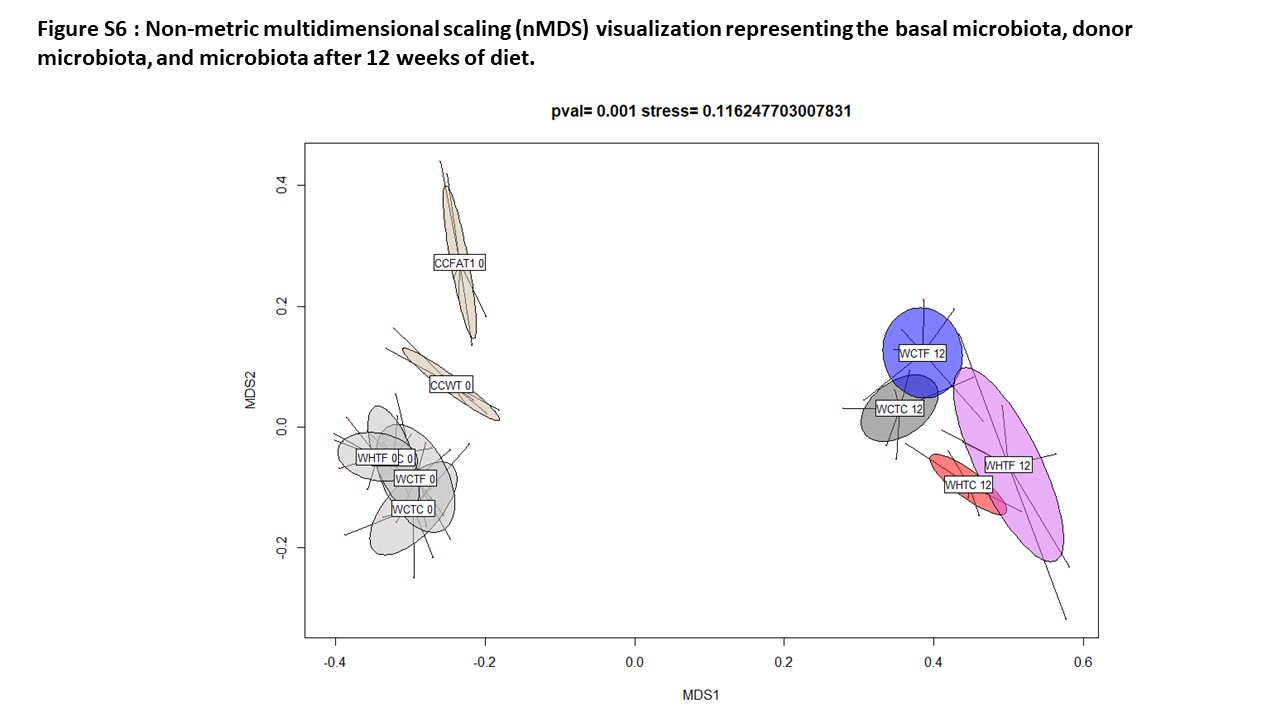

Supplement: Supplemental Material [file KGMI_A_2356270_SM0177.zip › FIGURE S6.jpg]

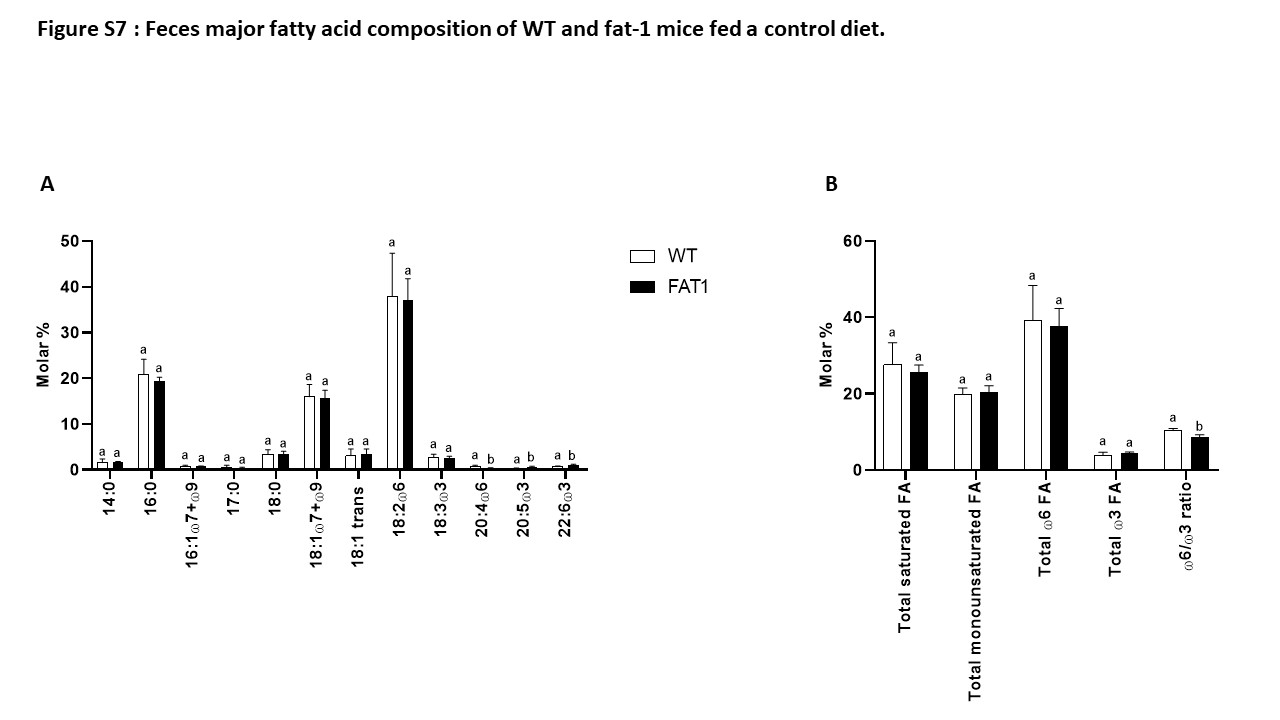

Supplement: Supplemental Material [file KGMI_A_2356270_SM0177.zip › FIGURE S7.jpg]
